# *Bambusananus cuihuashanensis*, a new bamboo-feeding leafhopper species of Athysanini (Hemiptera, Cicadellidae, Deltocephalinae) from Shaanxi, China

**DOI:** 10.3897/zookeys.341.5930

**Published:** 2013-10-08

**Authors:** Lin Yang, Xiang-Sheng Chen

**Affiliations:** 1 Institute of Entomology / The Provincial Key Laboratory for Agricultural Pest Management of Mountainous Region, Guizhou University, Guiyang, Guizhou, 550025, P.R. China

**Keywords:** Bamboo leafhopper, Cicadomorpha, distribution, Homoptera, taxonomy

## Abstract

*Bambusananus cuihuashanensis* **sp. n.** (Hemiptera: Cicadellidae: Deltocephalinae: Athysanini), a new bamboo-feeding leafhopper species, is described and illustrated from Shaanxi Province of China. Checklist, host plants and distribution for each species of *Bambusananus* is given along with a key to all known species.

## Introduction

The leafhopper tribe, Athysanini, was established by Van Duzee in 1892 and is the largest tribe of Deltocephalinae, including 228 genera and 1123 species (Zahniser and Dietrich 2013). Because this is such a large tribe, it is difficult or impossible to provide a set of characters that will easily diagnose it. There is substantial morphological diversity in the group, but most members have a Y-shaped connective and lack the distinctive features of other tribes (Zahniser and Dietrich 2013). Athysanini can be found in nearly all terrestrial ecosystems. Athysanini feed on a wide variety of eudicots and some species occasionally feed on grasses or sedges.

The leafhopper genus *Bambusananus* (Deltocephalinae: Athysanini) was established by Li et al. (2011) with the species *Bambusananus furcatus* Li & Xing, 2011, from Guizhou Province of China as its type species, and three new combinations: *Bambusananus binotatus* (Li & Dai, 2003), *Bambusananus bipunctatus* (Li, 1999) and *Bambusananus maculipennis* (Li & Wang, 1993) were proposed at the same time. Yang and Chen (2012) described one new species and summarized information on host plants and geographical distribution of the known species. Up to now, five species were recognised within this genus: *Bambusananus bipunctatus* (Li, 1999), *Bambusananus maculipennis* (Li & Wang, 1993), *Bambusananus furcatus* Li & Xing, 2011, *Bambusananus lii* (McKamey & Hicks, 2007) and *Bambusananus yangae* Xing & Chen, 2013 (Xing and Chen 2013). All members of the genus feed exclusively on Bambusoideae (Yang and Chen, 2012) and are currently known only from southern mainland China and Taiwan (Fig. 12).

During on-going studies on species biodiversity of the bamboo-feeding leafhoppers in China, several specimens belonging to an undescribed species of *Bambusananus* were found. The purpose of this paper is to describe this new species, to summarize information on host plants and geographical distribution of the known species and to provide a key to species in the genus.

## Materials and methods

In the present paper, terminology follows Li et al. (2011) except that for leg chaetotaxy follows Zahniser and Dietrich (2013). Dry specimens were used for the descriptions and illustrations. External morphology was observed under a stereoscopic microscope and characters were measured with an ocular micrometer. Measurements are given in millimeters; body length is measured from the apex of the head to the apex of the forewing in repose. The genital segments of the examined specimens were macerated in 10% KOH, washed in water and transferred to glycerine. Illustrations of the specimens were made with a Leica MZ 12.5 stereomicroscope. Photographs of the types were taken with a Leica D-lux 3 digital camera. The digital images were then imported into Adobe Photoshop 8.0 for labeling and plate composition. The type specimens and material examined are deposited in the Institute of Entomology, Guizhou University, Guiyang, China (IEGU).

## Taxonomy

### Checklist, host plant and distribution of species of genus *Bambusananus*

1. *Bambusananus bipunctatus* (Li, 1999) (in Cai and Huang 1999)
  Host plant. Bamboo (*Indocalamus hirsutissimus* Z. P. Wang & P. X. Zhang and *Qiongzhuea communis* Hsueh & Yi) (Yang and Chen 2012).
  Distribution. China (Guizhou, Fujian and Sichuan) (Fig. 12).
2. *Bambusananus maculipennis* (Li & Wang, 1993)
  Host plant. Bamboo (*Chimonobambusa pachystachys* Hsuch & W. P. Zhang and *Qiongzhuea communis* Hsueh & Yi) (Yang and Chen 2012).
  Distribution. China (Guizhou) (Fig. 12).
3. *Bambusananus cuihuashanensis* sp. n.
  Host plant. Bamboo.
  Distribution. China (Shaanxi) (Fig. 12).
4. *Bambusananus furcatus* Li & Xing, 2011
  Host plant. Bamboo (*Chimonobambusa angustifolia* C. D. Chu & C. S. Chao) (Chen et al. 2012).
  Distribution. China (Guizhou) (Fig. 12).
5. *Bambusananus lii* (McKamey & Hicks, 2007)
  Host plant. Bamboo (Li et al. 2011).
  Distribution. China (Taiwan) (Fig. 12).
6. *Bambusananus yangae* Xing & Chen, 2013
  Host plant. Bamboo (*Indocalamus* sp.) (Yang and Chen 2012).
  Distribution. China (Guizhou) (Fig. 12).

### Key to species of *Bambusananus* Li & Xing (male)

**Table:** 

1	Upper area of frontoclypeus with a large black transverse marking	2
–	Upper area of frontoclypeus without above marking (Fig. 2)	3
2	Aedeagal shaft with appendages long, reaching to middle of aedeagus (Fig. 11)	*Bambusananus cuihuashanensis* sp. n.
–	Aedeagal shaft with appendages short, only reaching to apical one-fifth of aedeagus	*Bambusananus bipunctata* (Li)
3	Ventral processes of male pygofer curved dorsad	*Bambusananus maculipennis* (Li & Wang)
–	Ventral processes of male pygofer with apical half curved ventrad (Fig. 6)	4
4	Appendages of aedeagal shaft branched	*Bambusananus furcatus* Li & Xing
–	Appendages of aedeagal shaft not branched (Figs 10, 11)	5
5	Aedeagal appendages arising from middle of aedeagus, straight, with apex directed apically; ventral margin of aedeagus without any teeth	*Bambusananus yangae* Xing & Chen
–	Aedeagal appendages arising from apical 1/3 of aedeagus, curved, with apex directed basolaterally; ventral margin of aedeagus with a row of teeth	*Bambusananus lii* (McKamey & Hicks)

### Bambusananus cuihuashanensis sp. n.

http://zoobank.org/4B853422-EEC6-41EB-AD6B-B1570C4E92E2

http://species-id.net/wiki/Bambusananus_cuihuashanensis

Figs 1–11

#### Type material.

Holotype: ♂, **China:** Shaanxi, Xi’an, Cuihuashan (108°57'E, 34°10'N), on bamboo, 37 Aug. 2008, J.-D. Li; paratypes: 2 ♂♂, 4 ♀♀, same data as holotype.

#### Etymology.

The new species is named after its locality, Cuihuashan, Shaanxi Province, China.

#### Measurements.

Body length (from apex of vertex to tip of forewings): male 4.75–4.85 mm (N = 2); female 5.75–5.90 mm (N = 4); forewing length: male 3.95–4.05 mm (N = 2); female 5.00–5.15 mm (N = 4).

#### Coloration.

**Figures 1–11.**
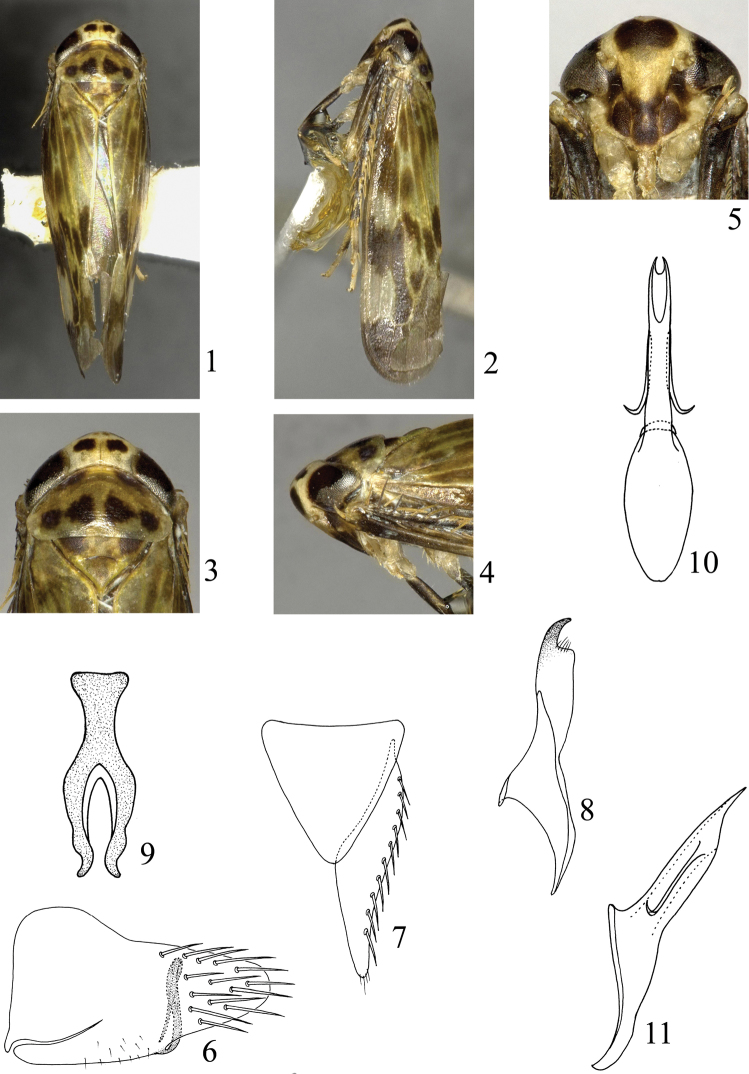
*Bambusananus cuihuashanensis* sp. n. **1** Male habitus, dorsal view **2** Male habitus, lateral view **3** Head and thorax, dorsal view **4** Head and thorax, lateral view **5** Head and thorax, ventral view **6** Male pygofer, lateral view **7** Genital valve and left subgenital plate, ventral view **8** Left style, dorsal view **9** Connective, dorsal view **10** Aedeagus, ventral view **11** Aedeagus, lateral view.

Crown (Fig. 1) pale yellowish white, two markings behind ocelli blackish brown. Eyes (Fig. 1) blackish brown, ocelli yellowish white. Frontoclypeus (Fig. 5) pale yellowish white, with lower area dark brown and a large kidney-shaped black marking at upper area; anteclypeus, lorums, genae with upper areas dark brown. Antennae (Fig. 5) pale yellowish brown. Pronotum (Figs 1, 3) pale yellowish brown, anterior areas with two dark brown markings, posterior areas with four blackish brown markings. Scutellum (Figs 1, 3) pale yellowish brown, with two brown markings basally. Forewing (Figs 1, 2) pale yellowish white to yellowish brown, veins yellowish white, with irregular blackish brown markings at median and posterior region. Thorax dark brown ventrally; legs brown to dark brown, except base of tarsus yellowish brown. Abdomen dark brown dorsally and ventrally, lateral margins of each segment pale yellowish white.

#### Head and thorax.

External features as in generic description. Crown shorter medially than width between eyes (0.54:1). Pronotum longer medially than crown (2.10:1). Scutellum shorter medially than pronotum (0.89:1). Forewing longer medially than width at widest part (3.38:1).

#### Male genitalia.

Male pygofer (Fig. 6) with basal half nearly quadrate, then narrowing to apex, dorsal margin slightly sinuate, ventral margin broadly curved, smooth, apical margin acute and rounded, with several macrosetae at apical area; ventral process slender and long, slightly widening at middle, narrowing apically, arising from inner side of ventral margin, produced dorsad, then abruptly strongly curved ventrally. Genital valve (Fig. 7) triangular, basal width slightly longer than median length (1.06:1). Subgenital plate (Fig. 7) moderately narrow, triangular, inner margin nearly straight, outer margin slightly concave, narrowing apically, apex acute and rounded, with row of macrosetae laterally. Style (Fig. 8) broad at base, abruptly narrowing subapically and curved hook-like. Aedeagus (Figs 10, 11) with shaft broad at middle, narrowing basally and apically, gonopore at apex, paired appendages slender and long, apex acute, arising from apical one-fourth, directed basally, then laterally. Connective (Fig. 9) Y-shaped, stem robust and arms well developed, stem slightly shorter than arm (0.79:1).

#### Host plant.

Bamboo.

#### Distribution.

**Figure 12.**
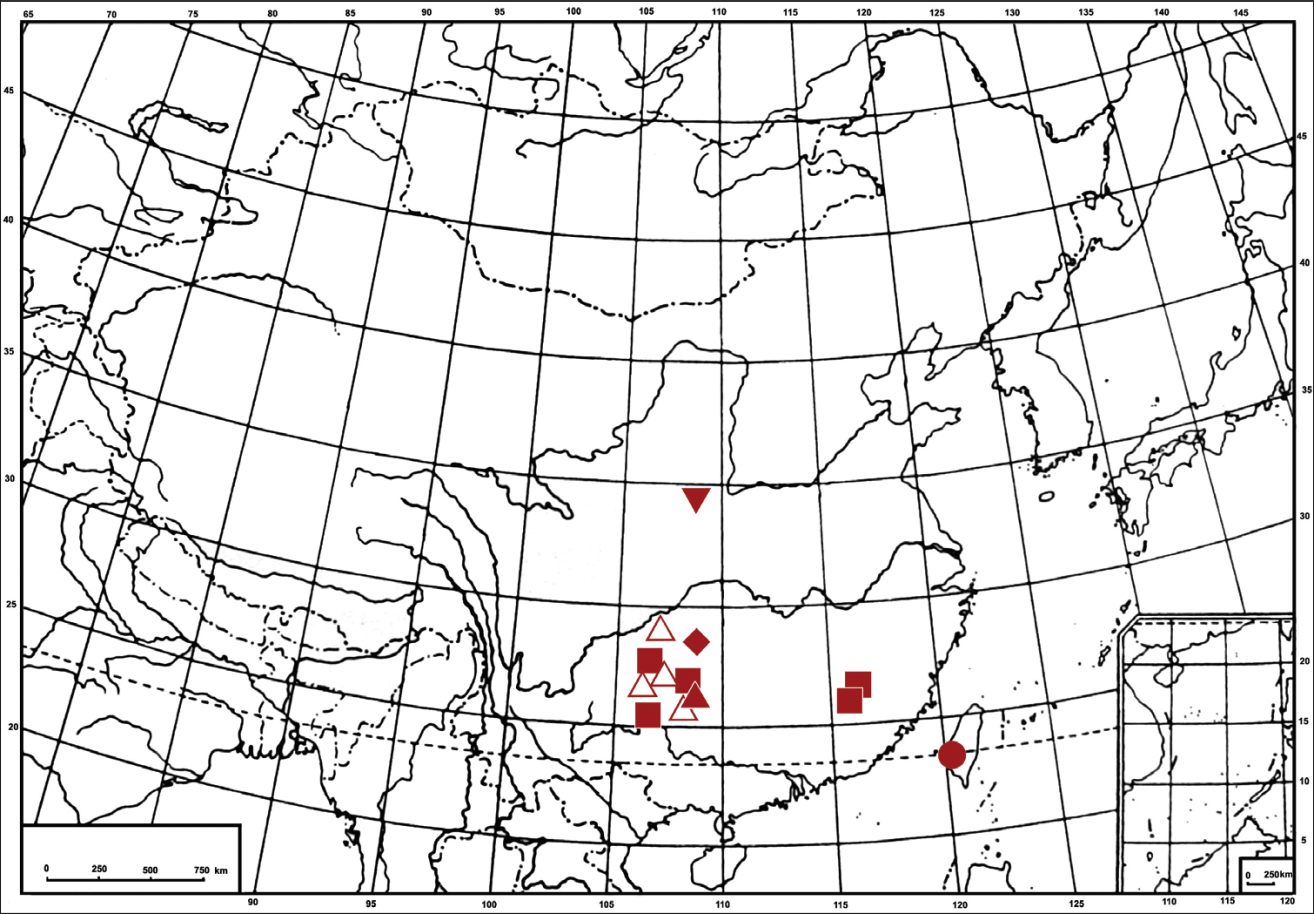
Geographic distribution of *Bambusananus* species: *Bambusananus bipunctatus* (Li) (■) *Bambusananus cuihuashanensis* sp. n.(▼) *Bambusananus furcatus* Li & Xing (▲) *Bambusananus lii* (McKamey & Hicks) (●) *Bambusananus maculipennis* (Li & Wang) (△) *Bambusananus yangae* Xing & Chen (◆).

China (Shaanxi) (Fig. 12).

#### Remarks.

This new species is similar to *Bambusananus bipunctatus* (Li, 1999) in general appearance, but can be distinguished by: pygofer in lateral view with ventral margin broadly smoothly rounded, without notch at base of ventral process (with notch in *Bambusananus bipunctatus*); connective with stem slightly shorter than arms (longer in *Bambusananus bipunctatus*); aedeagal shaft with appendages longer, in lateral view with apex reaching to middle of aedeagus (in *Bambusananus bipunctatus*, appendages shorter and apex only reaching to apical one-fifth of aedeagus). This new species is also similar to *Bambusananus lii* (McKamey & Hicks, 2007), but can be distinguished by: upper area of frontoclypeus with a large kidney-shaped black marking (lacking in *Bambusananus lii*); ventral margin of pygoger without any lobe (with a lobe near middle in *Bambusananus lii*); appendages of aedeagal shaft mostly straight basally, apex directed laterally (elbow-like and curved basally, apex directed caudad in *Bambusananus lii*); ventral margin of aedeagus without any teeth (with a row of teeth at middle in *Bambusananus lii*).

## Supplementary Material

XML Treatment for Bambusananus cuihuashanensis
